# Dexmedetomidine Mitigated NLRP3-Mediated Neuroinflammation *via* the Ubiquitin-Autophagy Pathway to Improve Perioperative Neurocognitive Disorder in Mice

**DOI:** 10.3389/fphar.2021.646265

**Published:** 2021-05-17

**Authors:** Lieliang Zhang, Fan Xiao, Jing Zhang, Xifeng Wang, Jun Ying, Gen Wei, Shoulin Chen, Xiangfei Huang, Wen Yu, Xing Liu, Qingcui Zheng, Guohai Xu, Shuchun Yu, Fuzhou Hua

**Affiliations:** ^1^ Department of Anesthesiology, The Second Affiliated Hosptial of Nanchang Univerisity, Nanchang, China; ^2^ Key Laboratory of Anesthesiology of Jiangxi Province, Nanchang, China; ^3^ Department of Anesthesiology, The First Affiliated Hosptial of Nanchang University, Nanchang, China

**Keywords:** dexmedetomidine, microglial, neuroinflammation, NLRP3 inflammasome, perioperative neurocognitive disorder

## Abstract

**Background:** Surgery and anesthesia-induced perioperative neurocognitive disorder (PND) are closely related to NOD-like receptors (NLR) family, pyrin domain containing 3 (NLRP3) inflammasome microglia inflammatory response. Inhibiting the occurrence of neuroinflammation is an important treatment method to improve postoperative delirium. Fewer NLRP3-targeting molecules are currently available in the clinic to reduce the incidence of postoperative delirium. Dexmedetomidine (DEX), an α2 adrenergic receptor agonist has been shown to have antioxidant and anti-inflammatory activities. The present study showed that DEX reduced the production of cleaved caspase1 (CASP1) and destroyed the NLRP3–PYD And CARD Domain Containing (PYCARD)–CASP1 complex assembly, thereby reducing the secretion of IL-1β interleukin beta (IL-1β). DEX promoted the autophagy process of microglia and reduced NLRP3 expression. More interestingly, it promoted the ubiquitination and degradation of NLRP3. Thus, this study demonstrated that DEX reduced NLRP3-mediated inflammation through the activation of the ubiquitin-autophagy pathway. This study provided a new mechanism for treating PND using DEX.

**Methods:** C57BL/6 mice were pre-administered DEX 3 days in advance, and an abdominal exploration model was used to establish a perioperative neurocognitive disorder model. The anti-inflammatory effect of DEX was explored *in vivo* by detecting NLRP3-CASP1/IL-1β protein expression and behavioral testing. Primary microglia were stimulated with lipopolysaccharide (LPS) and adenosine triphosphate (ATP) *in vitro*, the expression of CASP1 and IL-1β was detected in the supernatant of cells, and the expression of autophagy-related proteins microtubule-associated protein 1 light chain 3 beta (MAP1LC3B) and sequestosome 1 (SQSTM1) was examined in the cytoplasm. Meanwhile, Co-immunoprecipitation (Co-IP) was used to detect NLRP3 protein ubiquitination so as to clarify the new mechanism underlying the anti-inflammatory effect of DEX.

**Results:** Pre-administration of DEX reduced the protein expression of NLRP3, CASP1, and IL-1β in the hippocampus of mice induced by surgery and also improved the impairment of learning and memory ability. At the same time, DEX also effectively relieved the decrease in spine density of the hippocampal brain induced by surgery. DEX decreased the cleaved CASP1 expression, blocked the assembly of NLRP3–PYCARD–CASP1 complex, and also reduced the secretion of mature IL-1β *in vitro*. Mechanically, it accelerated the degradation of NLRP3 inflammasome via the autophagy–ubiquitin pathway and reduced the green fluorescent protein/red fluorescent protein MAP1LC3B ratio, which was comparable to the effect when using the autophagy activator rapamycin (Rapa). Furthermore, it increased the ubiquitination of NLRP3 after LPS plus ATP stimulated microglia.

**Conclusion:** DEX attenuated the hippocampal brain inflammation by promoting NLRP3 inflammasome degradation via the autophagy–ubiquitin pathway, thus improving cognitive impairment in mice.

## Introduction

Perioperative neurocognitive disorder is a severe complication following surgery and anesthesia in elderly patients. It is characterized by a decline in cognitive functions, such as attention, memory, and processing ability. Clinically, PND is usually accompanied by postoperative delirium, disruption of the blood–brain barrier, synapse damage, increased complications, and mortality of patients (Alam et al., 2018). the increase in the world’s population and the extension of human lifespan; hence, it seems important to effectively reduce the postoperative sequelae of perioperative patients (Kotekar et al., 2018). The underlying mechanism of PND remains complex and largely unknown, but the critical role of neuroinflammation in the development of PND has been proved. For example, the levels of serum inflammatory factors interleukin (IL)-6, tumor necrosis factor-α (TNF-α), and interleukin (IL)-18, increased in people aged more than 65 years (Pedersen et al., 2003; Ferrucci et al., 2005). A meta-analysis indicated that a cyclooxygenase inhibitor parecoxib improved early PND by reducing the release of local inflammatory factors (Huang et al., 2020). Further research on the mechanism of inflammatory response and aging and cognitive damage caused by surgery and anesthesia was an important breakthrough to solve this problem (Safavynia and Goldstein, 2018). However, despite tremendous efforts, various inflammation-targeting chemicals, such as IL-1β, neutralized antibodies, and none of the CASP1 inhibitors induced the expected clinical benefit from recent high-profile clinical trials (Dinarello et al., 2012; Lee et al., 2018). Given that the pathological mechanism of PND is complex, therapies that target the central nervous system’s inflammatory response are urgently needed.

Inflammasomes are critical mediators of CASP1 activation and IL-1β maturation, including NLR family pyrin domain containing 1 (NLRP1), absent in melanoma 2 (AIM2), NLR family pyrin domain containing 2 (NLRP2), NLRP3, and NLR family CARD domain containing 4 (NLRC4). The activation of inflammasome is related to some metabolic and neurodegenerative diseases, such as type 2 diabetes (T2D), Parkinson's disease (PD), Alzheimer’s disease (AD) (Mattson and Arumugam, 2018; Prattichizzo et al., 2018), as well as cognitive impairment induced by surgery and anesthesia. Several inflammasomes have been considered to be the main executor of mediating inflammatory responses in the central nervous system (CNS). NLRP3 inflammasome is most related to aging-associated diseases and injuries (Ferrucci and Fabbri, 2018; Tejera et al., 2019; Canadas-Lozano et al., 2020). It can be activated by different extracellular risk-related and pathogen-related molecular patterns, such as extracellular ATP, excess glucose, ceramides, amyloids, urate, and cholesterol crystals. Many mechanisms have been identified to inhibit NLRP3 inflammatory signaling in multiple steps (Kimura et al., 2017; Cordero et al., 2018). Among these, cell autophagy can promote the inactivation or selective elimination of harmful substrates or secondary substances, such as clearance of impaired mitochondria (Agyemang et al., 2015; Dan Dunn et al., 2015). Increasing evidence suggested that NLRP3 and autophagy pathways were linked through mutual regulation. However, autophagy-dependent degradation mechanisms remain still undefined.

Among the many steps that regulate NLRP3 activation, post-transcriptional modifications (PTMs), including ubiquitination and regulation, are particularly important and represent a new strategy. Research reports indicated that deubiquitination led to the activation of NLRP3 inflammasomes in mice and humans (Py et al., 2013; Guo et al., 2016), proving that NLRP3 ubiquitination was a key regulator of inflammatory responses. Although many NLRP3 regulations and networks were studied, the relationship between NLRP3 and ubiquitination was still poorly understood. Therefore, a better understanding of the mutual regulation of NLRP3 inflammasomes and PTMs is important for treating chronic neuroinflammation.

DEX is an α2 adrenergic receptor agonist with sedative, anxiolytic, and sympathetic nerve suppression effects and a positive therapeutic effect on inflammation in clinical trials (Demiri et al., 2019; Flanders et al., 2019). Also, the anti-inflammatory effect of DEX has been confirmed in animal experiments (Sanders et al., 2009; Hu et al., 2018). Recent research reports also pointed out that DEX exerted a protective effect against lung injury caused by intestinal ischemia–reperfusion in rats, possibly due to the upregulation of the CB2 receptor, followed by the activation of the phosphoinositide 3-kinase (PI3K)/Serine/Threonine Kinase (Akt) pathway (Chen et al., 2020). In addition, the findings showed that the cognitive improvement effect of Dex on traumatic brain injury was related to its inhibition of NLRP3 inflammasome activity in the hippocampus (Zheng et al., 2018). On the contrary, DEX improves lung function by promoting inflammation resolution in patients undergoing totally thoracoscopic cardiac surgery (Cui et al., 2020). However, in the PND mouse model, the mechanism of action of DEX is still poorly understood, and its anti-inflammatory mechanism needs to be explored. Further clinical evidence is needed to understand the underlying mechanisms.

In this study, the effect of DEX on surgery-induced inflammation and cognitive impairment by minimizing NLRP3 activation was confirmed in mice and cellular models. The data demonstrated that DEX reduced NLRP3 activation through the ubiquitin-autophagy pathway of NLRP3 protein. DEX regulated multiple targets, linking autophagy promotion and inflammation inhibition for PND prevention.

## Materials and Methods

### Animals

Male mice 12 months of age were used in this study referred to previous published papers and the groups of mice were randomly assigned (Wen et al., 2020). C57BL/6J mice (male, 12 months old) were obtained from Medical Animal Experiment Center of Nanchang University. They were bred and maintained in the Animal Resource Center of the Faculty of Nanchang University. They had free access to food and water in a room with an ambient temperature of 22 ± 2°C and a 12:12–h light/dark cycle. They were fasted for 12 h before surgery. Neonatal mice were used within 3 days. All animal procedures were performed in strict accordance with the Guide for the Care and Use of Laboratory Animals (National Institutes of Health, the United States) and the guideline of the Institutional Animal Care and Use Committee of Nanchang University.

### Construction of PND Models and Animal Drug Treatment

Exploratory laparotomy was performed to establish a postoperative delirium and *PND* model under aseptic conditions with isoflurane anesthesia, as described previously (Peng et al., 2016; Wen et al., 2020). Briefly, the mice were anesthetized with 1.8% isoflurane and oxygen at 2 L/min in an induction chamber for 30 min and then fixed supine on the operating table (Zuo et al., 2020). After disinfection of the abdomen, a sterile hole towel was spread. An incision of about 1.5 cm was made in the middle of the abdomen. The spleen, stomach, and duodenal organs were treated with caution following the principles of sterility to avoid damaging the organs of mice. The surgery was performed for about 30 min. The surgical incision was sterilized with iodophor and sutured. The mice were placed in a temperature-stable environment. After the mice woke up, they were placed in their original cages. DEX was administered (10, 20, and 30 μg/kg, intraperitoneally) once a day for 3 days before surgery. An incision of about 1.5 cm was made in the middle of the abdomen and stitched as soon as possible in the sham control group.

### Chemicals and Reagents

LPS (L2630), 3-MA (M-9281), Dexmedetomidine (SML-0956), MG-132 (C-2211), and rapamycin (R8781) were obtained from Sigma–Aldrich. ATP (S1985), Bafilomycin A1 (S1413), MCC950 (S7809), and LY294002 (S1105) were purchased from Selleck. Green fluorescent protein (GFP)-red fluorescent protein (RFP) MAP1LC3B double fluorescent adenovirus was obtained from HANBIO (HB-AP2100001, Shanghai, China). Hoechst (33342) was obtained from ThermoFisher. The primary antibodies for immunoblotting detection were as follows: NLRP1 (Cell Signaling Technology, 4990S, 1:1,000); NALP2 (Abcam, ab36850.1:1,000); NLRP3 (AdipoGen, AG-20B-0014-C100,1:1,000); NLRC4 (Abcam, ab201792, 1:1,000); AIM-2 (Santa Cruz, sc-137967, 1:1,000); Caspase1 (AdipoGen, AG-20B-0042, 1:1,000), IL-1β (R&D,AF-401-NA, 1:1,000), PSD-95 (Cell Signaling Technology, 3450, 1:1,000), ionized calcium-binding adaptor molecule-1 (IBA-1) (Wako, 019-19741, 1:1,000), BCL2-associated X (BAX) (Cell Signaling Technology, 14796, 1:1,000), BCL-2 (Cell Signaling Technology, 3498, 1:1,000), apoptosis-associated speck-like protein (ASC) (Santa Cruz, sc-22514, 1:1000), MAP1LC3B (Cell Signaling Technology, 2775, 1:1,000), Sequestosome 1 (SQSTM1) (Cell Signaling Technology, 5114s, 1:1,000), Autophagy related 5 (ATG5) (Signalway Antibody, 44254-4, 1:1,000), ATG7 (Signalway Antibody, 38148, 1:1,000), Ubiquitin (Abcam, ab7780, 1:1,000), and GAPDH (Santa Cruz, sc-32233,1:3,000). The secondary antibodies were obtained from Invitrogen (A16124, 32230, and A11058,1:3,000). Specific primary antibodies for immunofluorescence staining were as follows: glial fibrillary acidic protein (GFAP) (Abcam, ab7260, 1:1,000) and MAP-2 (Abcam, ab5392,1:500). The information on NLRP3 (1:500), Ubiquitin (1:500), and IBA-1 (1:500) antibodies was consistent with previous information. Fluorescence-specific secondary antibodies were obtained from Invitrogen (A11008 and A21422,1:1,000). The antibodies used for immunohistochemical staining were the same as those for immunoblotting.

### Immunoblotting

The cells and the hippocampal tissues were lyzed in the RIPA lysis buffer (Cell Signaling Technology) plus protease inhibitor (Halt Protease Inhibitor Single-Use Cocktail, Thermo Fisher Scientific). The protein content was detected using the BCA Protein Assay Kit (Beyotime). The proteins were separated by SDS-polyacrylamide gel electrophoresis and transferred to a PVDF (Millipore, IPVH00010) membrane activated with methanol, After blocking with 5% milk at room temperature for 1 h, PVDF membranes were incubated with various specific primary antibodies as described earlier in antibody dilution (NCM Universal Antibody Diluent, New Cell and Molecular Biotech Co., Ltd.) at 4°C overnight. The PVDF membrane was washed with TBST containing Tris-HCl, NaCl, and Tween 20, and incubated with HRP-conjugated secondary antibody. After incubation for 1 h at room temperature, the proteins were visualized and detected using BeyoECL Moon (Beyotime) and analyzed with an ImageQuant LAS 4000 imaging system (GE Healthcare, PA, United States).

### The Hippocampal Tissue Immunofluorescence Staining

The brain slices were washed three times with phosphate-buffered saline (PBS) and then incubated with 0.3% Triton X-100 in PBS supplemented with 5% bovine serum albumin (BSA) for 1 h at room temperature. Then, the brain slices were incubated with the specific primary antibody at 4°C overnight. After incubating with the fluorescent secondary antibody at room temperature the next day, the brain slices were placed on the slide and observed under a stereomicroscope (Olympus, Tokyo, Japan).

### Immunohistochemical Analysis

The hippocampal tissue slices were washed three times with PBS, followed by 3% H_2_O_2_, for 15 min to quench the endogenous peroxidase activity, and then incubated with 0.3% Triton X-100 in PBS supplemented with 5% BSA for 1 h at room temperature. The brain slices were incubated with the specific primary antibody at 4°C overnight. After incubating the slices with the secondary antibody the next day, they were incubated with diaminobenzidine for 5 min. PBS was used to stop the diaminobenzidine color reaction. The brain slices were dehydrated in alcohol and xylene and observed under a stereomicroscope (Olympus). The proportion of positive area was calculated using ImageJ software.

### Golgi-Cox Staining

Experimental method referring to previous studies (Li et al., 2021), after the mice were decapitated under deep anesthesia, the brains were quickly removed and washed with distilled water. The hippocampal tissue was immersed in the A and B impregnation solution (#PK401, FD NeuroTechnologies, MD, United States) and stored at room temperature for 14 days in the dark. Next, the brain tissue was transferred to the C solution and stored at room temperature in the dark for 7 days. Finally, it was embedded with the OCT glue to make a brain slice of about 100–200 μm, which was pasted on a glass slide containing C solution, treated with gelatin, and stored in the dark. After dyeing with D and E solution, the slices were dehydrated in alcohol and xylene and observed under a stereomicroscope (Olympus). The pictures taken were analyzed with the NeuroJ plugin in ImageJ software.

### Animal Behavior Experiment

#### Y- Maze

Y-maze was performed referring to literature concerned (Huang et al., 2015). Y maze consists of three long, wide, and high arms of 34, 10, and 10 cm, which are the starting arm, novel arm, and other arm, respectively. The experimental animals were placed in the test room to adapt to the environment 24 h before the experiment. At the beginning of the test, the novel arm was blocked with a baffle, and the mouse was put in from the starting arm. The mouse movement track was recorded after 5 min of free exploration in the starting arm and other arms. One hour later, the mice were placed in the starting arm, the novel arm was opened, the exploration was performed for 5 min, and the mouse movement track was recorded. The function of short-term memory in mice was measured by dividing the time spent in exploring the novel arm by the total time.

### New Object Recognition

New object recognition experiments are used to evaluate the ability of mice to recognize new objects in the environment. First, two identical objects were put in an autonomous mobile box (40 × 27 × 20 cm^3^), close to the sides of the box, and then the experimental animals were put into the box. The mice were allowed to explore freely for 5 min, and the time spent exploring each object was recorded (exploration was defined as sniffing or touching the object in a radius of 0–2 cm with its nose). After 5 min, one object was replaced with a novel one, the mice were allowed to explore for another 5 min, and the time spent by the animals exploring the novel and old objects was recorded.

### Primary Microglia Cultures

The hippocampus of neonatal mice aged 1–3 days were removed and separated from the meninges and basal ganglia. Tissues were dissociated with 0.25% trypase (Amresco, OH, United States) at 37°C for 10 min and terminated using Dulbecco's Modified Eagle Medium/F-12 medium (Gibco, Thermo Fisher Scientific) supplemented with 10% fetal bovine serum (FBS) (Gibco, Thermo Fisher Scientific) and 1% penicillin/streptomycin. The cells were plated on 25 cm^2^ flasks (Thermo Fisher Scientific) at a density of 3 × 10^6^ cells/mL. After 1 day, the culture medium was replaced with the fresh medium every 3 days, for about 10–12 days. The cells were shaken vigorously, and the culture medium was aspirated and transferred to a centrifuge tube. The culture medium was centrifuged at room temperature at 1,500 rpm for 10 min. The resuspended microglia were plated at a density of 5 × 10^5^ cells/mL in a 12-well plate pre-plated with polylysine.

### Cytotoxic Activity Test

Primary microglial cells were seeded in a 96-well plate. After the microglia were iron-walled overnight, different concentrations of DEX were added. Three replicates of each group were used, and 10 μl of cell counting kit 8 reagent (Bimake) was added to each well. Next, the cells were incubated for another 2 h until the color turned orange. The 96-well plate was placed in a full-wavelength microplate reader (Thermo Fisher Scientific), and light absorption at 450 nm was detected. The degree of cell proliferation and toxicity were calculated.

### Primary Neuron Culture

Cell culture was performed referring to literature concerned (Wei et al., 2020).16 day pregnant mice were sacrificed and E16 embryos were dissected out in 1X PBS, and the meninges and basal ganglia were removed under a microscope. Tissues were digested with 0.025% trypsin for 20 min at 37°C and terminated with 10% FBS. The cell suspension was passed through a 40 μm cell sieve and centrifuged for 5 min at 1000 *g*. The cell pellet was resuspended in neurobasal medium (Gibco, Thermo Fisher Scientific) supplemented with 2% B-27 (Gibco, Thermo Fisher Scientific) and 0.5 mM L-glutamic acid. The cells were seeded on a plate pre-coated with poly-D-lysine hydrobromide (Sigma–Aldrich). The neuronal cell culture could be used for experiments until 7 days, and fresh media needed to be replaced every 3 days.

### Cell Immunofluorescence Staining

The cell slides in the 24-well plate were washed three times with 0.1 M PBS and fixed with 4% paraformaldehyde for 15 min. The paraformaldehyde was aspirated, washed with PBS, and blocked with PBS containing 5% BSA for 1 h. Then, the cell slide was incubated with the specific primary antibody at 4°C overnight, and the fluorescent secondary antibody was incubated for 1 h at room temperature the next day. After aspirating the secondary antibody and covering the cell slide with a glass slide, the cells were observed under the stereomicroscope (Olympus).

### Flow Cytometry Analysis

The apoptosis of cells was detected using an AV-PI kit, following the manufacturer’s protocols (Vazyme, Annexin V–FITC/PI Apoptosis Detection Kit). The cells were incubated with AV-PI for 10 min, washed with PBS, and then analyzed using a Guava easyCyte System 8 (Millipore 25801, CA, United States).

### Cell Supernatant Extraction

The collected 1 ml of the cell supernatant was centrifuged at 13,000 *g* for 5 min, and 600 µl of it was carefully transferred into another EP tube. An equal volume of pre-cooled methanol and one-fourth volume of chloroform were added to the supernatant, quickly vortexed, and shaken. Then, the supernatant was centrifuged at 13,000 rpm for 5 min. The supernatant was discarded carefully. Subsequently, 500 µl of pre-cooled methanol was added to the pellet. The liquid in the EP tube was quickly shaken and centrifuged at 13,000 rpm for 5 min, and the supernatant was discarded carefully. The centrifuge tubes with cell pellet were placed in an oven at 37°C for 5 min, and then 40 µl of 2.5 × loading buffer was added to dissolve the protein. After boiling at 95°C for 5 min, the precipitate was used for immunoblotting.

### Co-Immunoprecipitation

Mouse primary microglia were lyzed in the buffer (Cell Signaling Technology). The proteins were immunoprecipitated with specific primary antibodies (1 μg antibody per 100 μg of total protein), followed by incubation with G Agarose (Santa Cruz Biotechnology) at 4°C overnight. Proteins were eluted from the beads prepared for Western blot analysis.

### Tandem Fluorescent-mRFP–GFP–MAP1LC3B–Adenovirus Transduction of Microglia

The primary microglia were transfected with a tandem fluorescent mRFP- GFP-MAP1LC3B adenovirus (HanBio, HB-AP2100001) following the manufacturer’s protocol. The GFP green fluorescence and RFP red fluorescence of cells indicated the hindrance and smoothness of the autophagy process. Six fields of view in each group of cells were chosen for detection. After the cells were processed, they were fixed with 4% polymethanol, rinsed with PBS, stained with Hoechst, and photographed. The ratio of green fluorescent dots to red fluorescent dots was calculated to reflect the process of autophagy.

### Statistical Analysis

All data were expressed as mean ± SEM and analyzed in a blind way using the Student *t* test, one-way analysis of variance (ANOVA), or two-way ANOVA. In all studies, *n* represents the number of samples per group. A *p* value < 0.05 indicated a statistically significant difference.

## Results

### NLRP3 Activation in Microglia Was Associated With Surgery-Induced Inflammation

**FIGURE 1:**
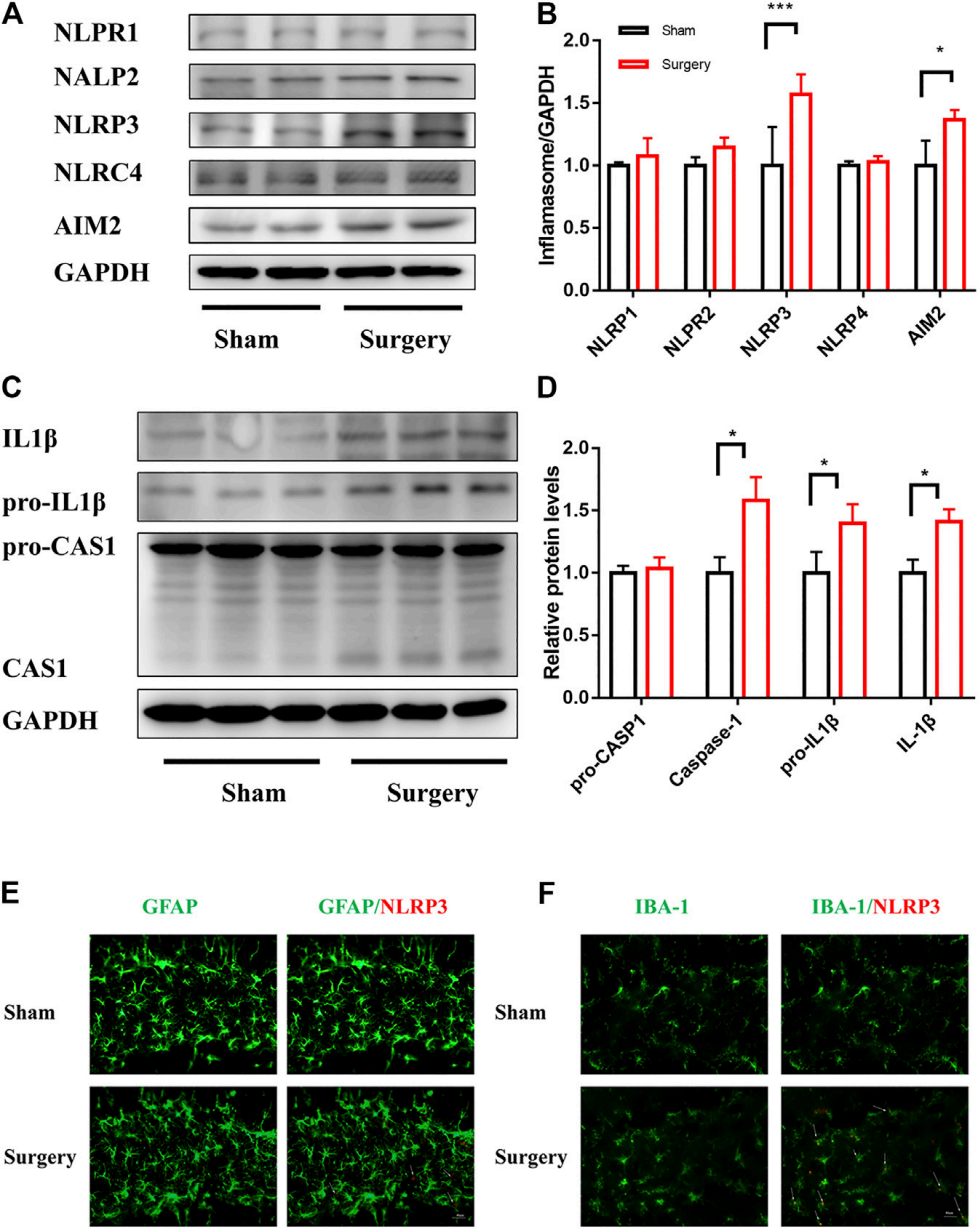
NLRP3 activation in microglia was associated with surgery-induced inflammation. NLRP3 activation in microglia was associated with surgery-induced inflammation. **(A)**: The expression levels of NLRP1, AIM2, NLRP2, NLRP3, and NLRC4 in the hippocampus were examined by Immunoblotting, and the statistical data are shown in **(B)** (means ± SEM, *n* = 3). **(C)**: pro-IL1β/IL1β,pro-CASP1/CASP1/CASPASE1 protein expression in the hippocampus were measured by Immunoblotting and the quantitative analysis data shown in **(D)** (means ± SEM, *n* = 3). **(E,F)** Immunofluorescence staining of IBA-1, GFAP and NLRP3 in the hippocampal dentategyrus brain region. *p < 0.05, **p < 0.01, ***p < 0.001 compared with the corresponding sham group, as determined by the Student’s *t*-test.

Neuroinflammation is one of the main pathological factors for most neurodegenerative diseases and acute surgical anesthesia stress (Bright et al., 2019). Recent studies indicated that inflammasome-mediated neuroinflammation was vital in surgical anesthesia stress–induced PND (Wei et al., 2019). Furthermore, some studies detected mature IL-1β levels in the peripheral blood, indicating a significant activation of inflammation in many diseases (Amdur et al., 2019). This study aimed to clarify the link between neuroinflammation and cognitive impairment caused by surgery and anesthesia. A PND model of mice was established for abdominal exploration. The hippocampal brain areas related to learning and cognition were evaluateds *in vivo.* The immunoblotting results showed that the expression levels of NLRP3 and AIM2, especially NLRP3 expression levels, in the hippocampus of mice significantly increased in the surgery group compared with the sham group (*p* < 0.05) (Figures 1A,B). However, the expression levels of NLRP1, NLRP2, and NLRC4 were not significantly different between the two groups. Similarly, CASP1 and IL-1β, the components of NLRP3 inflammasome in the hippocampus, had high expression levels (Figures 1C,D). Furthermore, the immunofluorescence staining was used to detect the expression of GFAP and IBA-1, which are the markers of astrocyte and microglia, to investigate whether NLRP3 expression on the glia of the hippocampal dentate gyrus was activated by surgery. As shown in Figures 1E,F, NLRP3 on microglia was significantly activated in the surgery group, and the degree of co-expression of IBA-1 and NLRP3 was higher than that of GFAP and NLRP3. In summary, these results suggested that NLRP3 expression was related to a surgery-induced inflammatory response.

### DEX Mitigated Surgically Induced Nerve Damage and Reversed Cognitive Function in Mice

**FIGURE 2:**
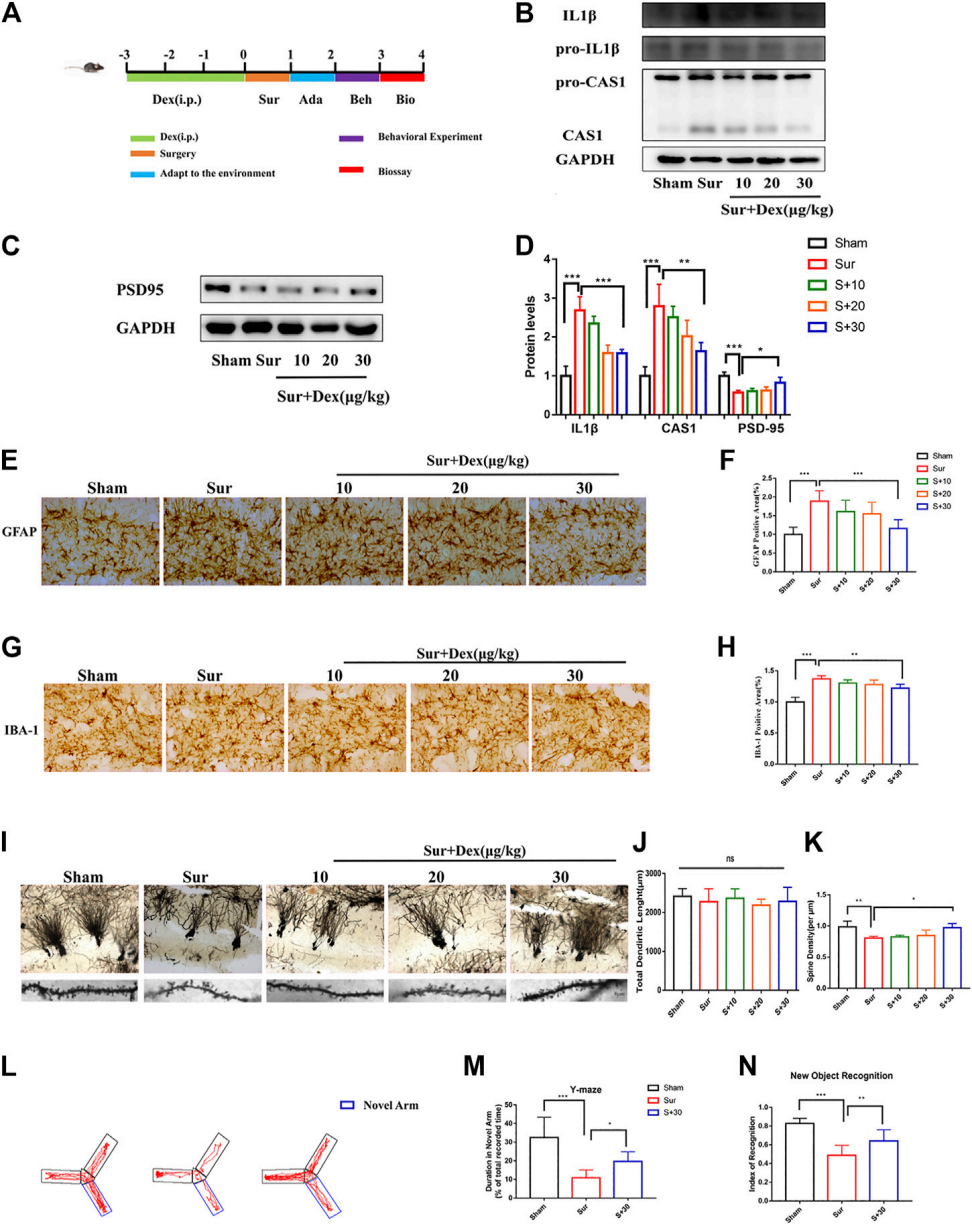
DEX mitigated surgically induced nerve damage and reversed cognitive function in mice. **(A)** Diagram of the experimental design, DEX was given once a day three days before the operation, and the first day after the operation was adapted to the environment of the behavioral test. The biological test was performed after the behavioral test. pro-IL1β/IL1β, pro-CASP1/CASP1 and PSD95 protein expression in the hippocampus were measured by Immunoblotting **(B,C)** and the quantitative analysis data shown in **(D)** (means ± SEM, *n* = 4). *p < 0.05, **p < 0.01, ***p < 0.001 compared with the corresponding group, as determined by the Student’s t-test. The expression of GFAP and IBA-1 were analyzed by immunochemistry in the hippocampal dentategyrus brain region **(E–G)**, and the statistical data are shown in graph **(F–H)** (means ± SEM, *n* = 6). Scale bars = 100 μm. Quantification of the positive areas using ImageJ software. *p < 0.05, **p < 0.01, ***p < 0.001 compared with the corresponding group, as determined by the Student’s *t*-test. The hippocampus of the sections were examined by Golgi-Cox Staining **(I)**, the pictures taken were analyzed with the NeuroJ plugin in ImageJ software to calculate the total length of dendrites and the density of synaptic spines (**J,K**, *n* = 6). *p < 0.05, **p < 0.01, ***p < 0.001 compared with the corresponding group, as determined by the Student’s t-test. Y-maze and new object recognition were tested of mice at the end of the experiment (means ± SEM, *n* = 10), the Y-maze trace of the mice were shown in **(L)**, and the quantitative analysis data shown in **(M–O)**. *p < 0.05, **p < 0.01, ***p < 0.001 compared with the corresponding group, as determined by the Student’s *t*-test.

Several animal and clinical studies have shown significant anti-inflammatory effects of DEX. Given that neuroinflammation is an important factor leading to PND, a PND model and a pre-administration group of DEX were established to explore the effect of DEX on inflammatory response and cognitive function in mice. As shown in Figure 2A, DEX was administered (30 μg/kg, intraperitoneally) once a day for 3 days before the surgery. The immunoblotting results showed that the expression of CASP1/IL-1β in the hippocampal tissue significantly increased in the surgical group compared with the sham group. DEX treatment attenuated the protein expression in a dose-dependent manner (Figure 2B). Surgical stress reduced the expression of postsynaptic density-95 (PSD-95), and the pre-protection of Dex can increase the expression of PSD-95. (Figures 2C,D). The inflammatory reactions were usually accompanied by the activation of glial cells. The expression of GFAP and IBA-1 was detected to investigate whether DEX had an effect on glia activation induced by surgery and anesthesia. Significant glial cell activation was observed in the surgery group, but DEX (30 μg/kg) administration significantly attenuated the activation of both astrocytes and microglia (Figures 2E,H). Golgi-Cox staining was used to examine the density of dendritic spines in the hippocampal region, which was thought to be closely related to learning and memory. The spine density was lower in the surgery group than in the sham group. DEX (30 μg/kg) treatment improved the decrease in spine density induced by surgery. However, the total dendritic length did not differ between these groups (Figures 2I–K). Finally, the behavioral analysis of spatial learning memory was performed using Y maze and novel object recognition tests to assess whether the efficacy of DEX improved against cognitive impairment in mice. The behavioral results showed that the exploration time of the DEX pre-protected group (30 μg/kg) in the new arm was longer than sham group in the Y maze experiment. Similarly, the experimental data showed that DEX pre-protected group (30 μg/kg) of the time spent exploring new things was longer than sham group in the new object recognition (Fig. 2L–2N). Collectively, these results suggested that DEX mitigated surgery-induced inflammation and reversed cognitive dysfunction caused by anesthesia and surgery.

### DEX Reduced Microglia Activation and Attenuated Neuronal Cell Apoptosis *In Vitro*

**FIGURE 3:**
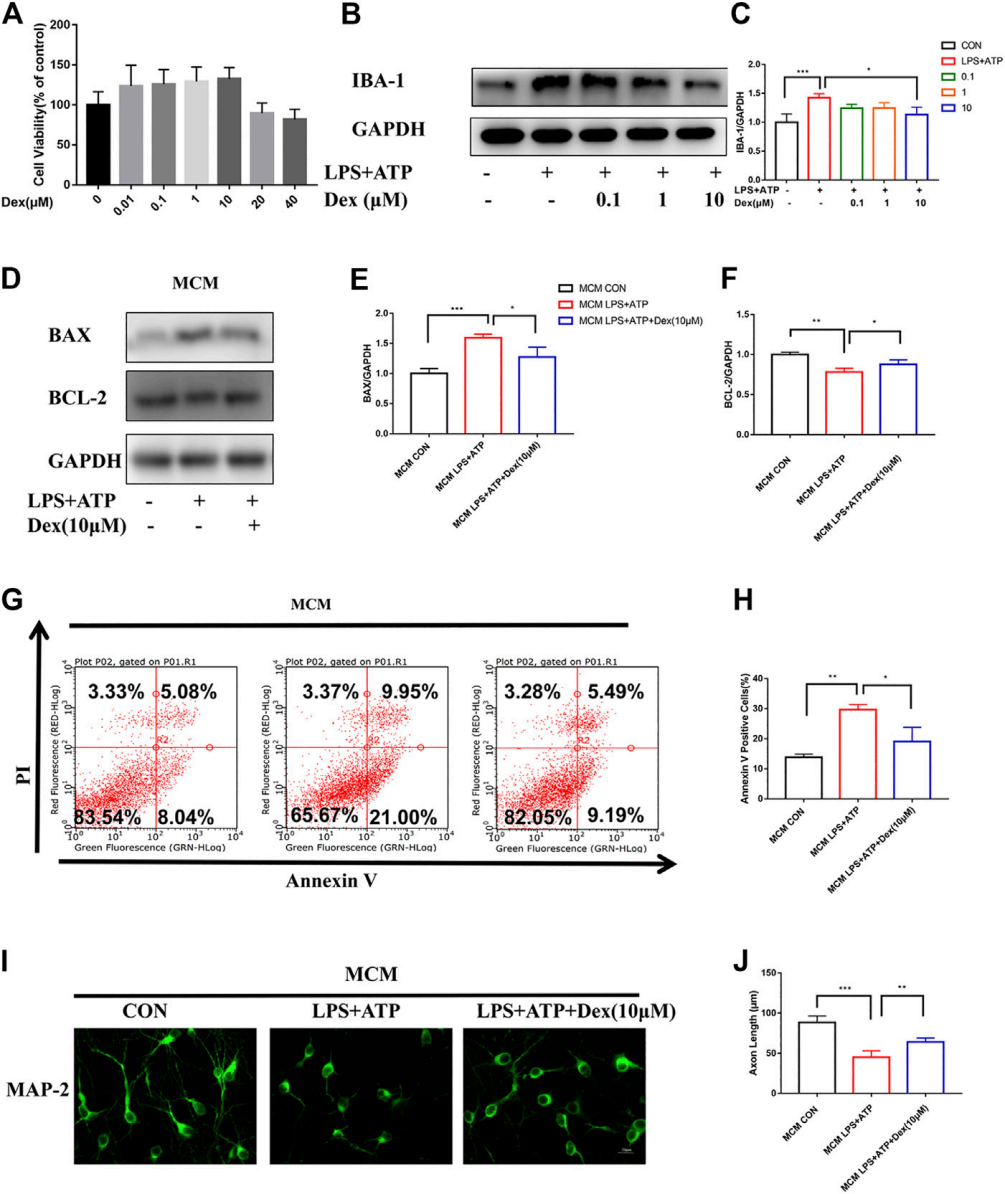
DEX reduced microglia activation and attenuated neuronal cell apoptosis *in vitro*. DEX reduces microglia activation and attenuates neuronal cell apoptosis *in vitro*. The primary microglia viability and toxicity test were examined by the CCK-8 kit. Each group had three replicates, and the test was repeated 4 times independently **(A)**. LPS-primed (100 ng/ml) primary microglia were treated with different doses of DEX for 5 h and then stimulated with ATP (5 mM) for 30 min. The expression of IBA-1 detected by Immunoblotting **(B)** and the statistical data shown in **(C)** (means ± SEM, *n* = 4). LPS-primed primary microglial cells were incubated with DEX for 5 h, then stimulated with ATP for 30 min.Microglia conditioned medium including without any treatment, treated with LPS (100 ng/ml, 5 h)plus ATP (5 mM, 30 min), and DEX (10 μM, 5 h) protected, MCM mixed with 2% B-27 Neurobasal medium (1:1) and incubated the cells for 24 h, primary neuronal cells were incubated with mix MCM separately, collected for Immunoblotting, immunofluorescence staining and cell flow analysis. Expression of apoptosis-related proteins BAX and Bcl-2 detected by Immunoblotting **(D)**, the statistical data are shown in **(E,F)** (means ± SEM, *n* = 4). Apoptosis indicators were detected using a cell flow cytometer **(G)**. Data analysis is shown in **(H)** (means ± SEM,n = 4). MAP-2 expression was stained by immunofluorescence and axon length was calculated by IMageJ software **(I)**, data analysis in **(J)** (means ± SEM, *n* = 4). All the above data analysis were presented as the means ± SEM,*p < 0.05, **p < 0.01, ***p < 0.001 compared with the corresponding group by Student’s *t*-test.

The results indicate that DEX could reduce surgically induced nerve damage caused by inflammation *in vivo*. Hence, the neuroprotective effect of DEX were further verified *in vitro*. First, the effect of DEX on microglia activation was examined in LPS- and ATP-primed mouse primary microglia after determining the optimal concentration of DEX through cell viability and cell proliferation experiments *in vitro* (Figure 3A). As shown in Figures 3B,C, the treatment of DEX blocked IBA-1 expression in a concentration-dependent manner. Therefore, a dose of 10 μM was chosen for subsequent experiments. Previous studies reported that activated microglia damaged neurons through multiple pathways: production of superoxide directly caused cell necrosis or apoptosis; release of matrix metalloproteinases caused hypoxic-ischemic neuronal damage, reducing nutrition; and production of protective brain-derived neurotrophic factors and insulin-like growth factors indirectly increased neuronal apoptosis. Therefore, the cells were incubated with microglial conditioned media (MCM) mixed with 2% B-27 neurobasal medium (1:1) for 24 h to investigate the neuroprotective effects of DEX. The proteins of primary neurons were detected by immunoblotting. The expression of apoptosis-related protein BAX (apoptosis regulator) was upregulated, and the expression of BCL-2 (apoptosis regulator) was downregulated after incubation with LPS and ATP, but this response was reversed in the DEX treatment group (Figures 3D–F). In addition, the Annexin V/PI staining indicated that the neurons incubated with LPS and ATP MCM promoted the occurrence of early apoptosis. However, DEX protected neurons by reducing the release of microglial inflammatory factors, thus minimizing the occurrence of early apoptosis (Figures 3G,H). Similarly, the results of microtubule-associated protein2 (MAP-2) immunofluorescence showed that the microglial inflammation induced by LPS and ATP shortened the axon length of neuronal cells, but the culture medium of microglial cells treated with DEX reduced neuronal damage and improved axon length (Figures 3I,J). Taken together, these results indicated that DEX reduced microglia activation and neuronal cell apoptosis *in vitro*.

### DEX Suppressed CASP1 and IL-1β Maturation by Interrupting NLRP3 Inflammasome Activation and Assembly

**FIGURE 4:**
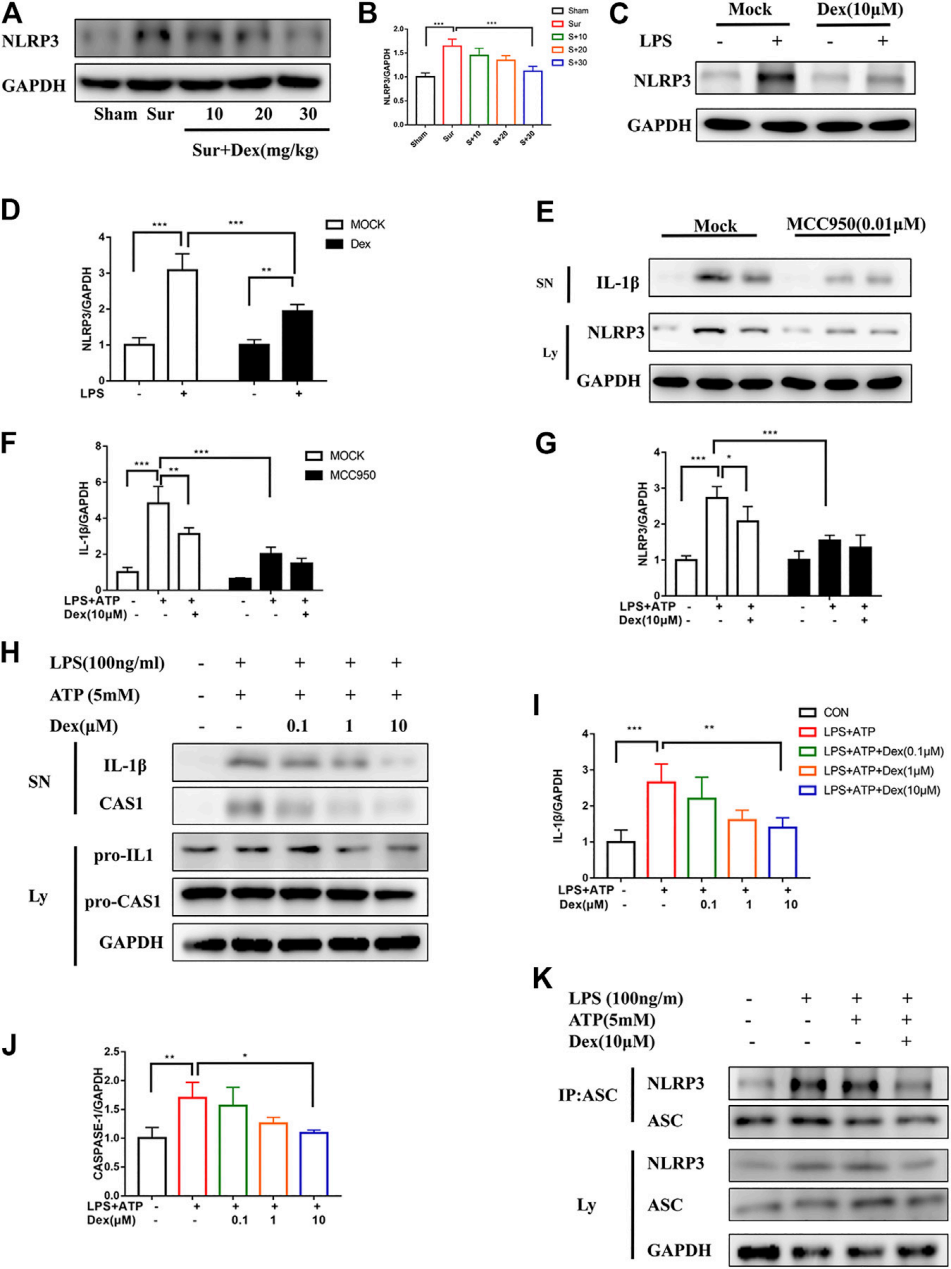
DEX suppressed CASP1 and IL-1β maturation by interrupting NLRP3 inflammasome activation and assembly. DEX suppressed CASP1 and IL-1β maturation by interrupting NLRP3 inflammasome activation and assembly. Protein NLRP3 in the hippocampus brain region of different groups of mice was detected by Immunoblotting **(A,B)** is the corresponding statistical analysis (means ± SEM, *n* = 4). LPS-primed primary microglial cells were incubated with DEX for 5 h, and NLRP3 expression was detected via Immunoblotting **(C)**, and the data shown in **(D)** (means ± SEM, *n* = 4). Primary microglial cells were treated as follows: MCC950 (0.01 μM) was administered 2 h in advance, LPS-primed primary microglial cells were incubated with DEX for 5 h and then ATP was gived for 30 min. The protein cell supernatant protein and cytosolic protein were collected for Immunoblotting to detect IL-1βand NLRP3 **(E)**. The data is shown in **(F,G)** (means ± SEM, *n* = 4). The expression of the pro-IL-1β/IL-1β, pro-CASP1/CASP1 examined by Immunoblotting **(H)**, the cells of the treatment is the same way as above. Protein expression analysis data were shown in **(I,J)** (means ± SEM, *n* = 4). The NLRP3-ASC interaction was analyzed by CO-IP and Immunoblotting **(K)**. All the above data analysis were presented as the means ± SEM, *p* < 0.05 is defined as statistically different, and the ns means no significance, *p < 0.05, **p < 0.01, ***p < 0.001 compared with the corresponding group. The data analysis in **(B,I,J)** was one-way ANOVA, data analysis in **(D,F,G)** was two-way ANOVA. SN, supernatant; Ly, lysate.

NLRP3 expression in the hippocampus was measured to investigate the mechanism underlying the neuroprotective effect of DEX. As shown in Figures 4A,B, the pre-administration of DEX reduced NLRP3 expression induced by surgery in a dose-dependent manner in a mouse model. At the same time, primary microglia were cultured and stimulated with LPS (100 ng/ml, 5 h) to verify the experimental results *in vitro*, which was the first activation signal of inflammation, prompting the expression of NLPR3 protein. Similarly, DEX attenuated the expression level of NLRP3 in primary microglia (Figures 4C,D). Considering the involvement of NLRP3 in inflammation, MCC950 (an inhibitor of NLRP3) was used to suppress NLRP3 expression in primary microglia. Also, LPS and ATP were administered to induce the activation and assembly of NLRP3 inflammasomes. IL-1β expression significantly reduced after interfering with NLRP3 expression compared with that in the group without MCC950 treatment; DEX reversed this effect (Figures 4E–G). NLRP3 inflammasome is composed of NLRP3 protein, pro-CASP1, and ASC, which are important in developing inflammation. Therefore, the present study further examined the effect of DEX on the expression of IL-1β and ASC after LPS and ATP stimulation. The results showed that DEX reduced CASP1 activation, IL1β maturation, and ASC oligomerization induced by LPS and ATP dose-dependently (Figures 4H–K). These results confirmed that DEX reduced the maturation of CASP1, production of IL-1β, and oligomerization of ASC, and then inhibited the activation and assembly of NLRP3 inflammasome.

### DEX Promoted NLRP3 Degradation via the Autophagy Pathway

**FIGURE 5:**
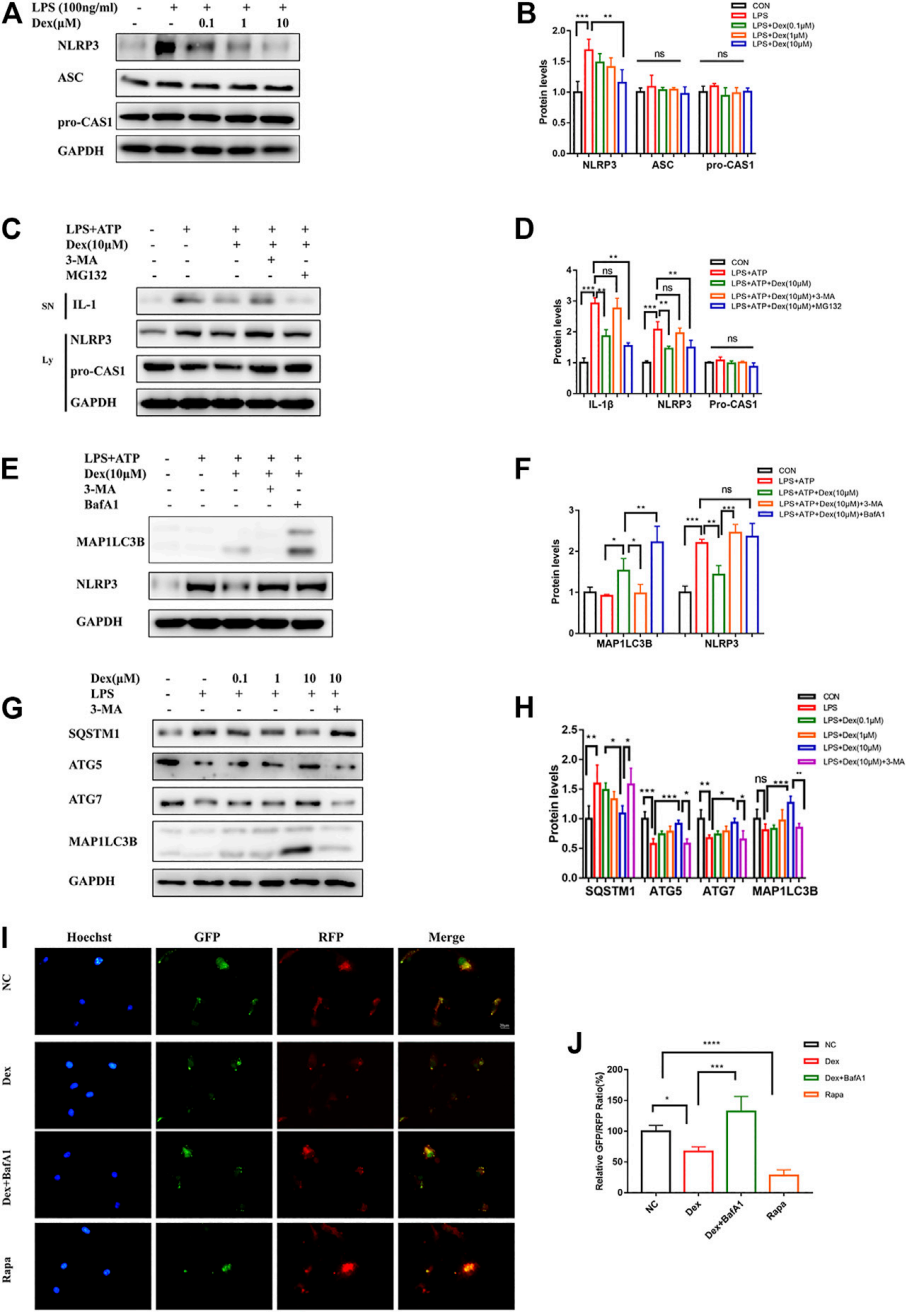
DEX promotes NLRP3 degradation via autophagy pathway. DEX promoted NLRP3 degradation via the autophagy pathway. The cells were treated in the same way as before, and the NLRP3,ASC,pro-CAS1 were detected via Western blot (**A,B**, means ± SEM, *n* = 4). LPS-primed primary microglial were cultured with MG-132 (10 μM), 3-MA (5 mM) or BafA1 (100 nM) for 1 h and then treated with DEX (10 μM) for 5 h, followed by a 30-min incubation with 5 mM ATP. The protein expression of the Supernatants (SN) (IL-1β) and cell extracts (lysate) (NLRP3,pro-CASP1, and MAP1LC3B) were analyzed by Immunoblotting (**C,E**, means ± SEM, *n* = 4). The corresponding data analysis were shown in **(D–F)** (means ± SEM, *n* = 4). The protein expression of SQSTM1, ATG5, ATG7, and MAP1LC3B-II after LPS stimulation were analyzed by western blot (**G,H**, means ± SEM, *n* = 4). Cells used for experiments 4 days after RFP-GFP-MAP1LC3B adenovirus infection. The cells were treated in the same way as before. The relatively GFP-RFP ratio were counted **(I)** and the analysis data were shown in **(J)** (means ± SEM, *n* = 4). All the above data analysis were presented as the means ± SEM, *p* < 0.05 is defined as statistically different, and the ns means no significance,*p < 0.05, **p < 0.01, ***p < 0.001 compared with the corresponding group. Data analysis method was one-way ANOVA.

To limit the dissipation in experimental animals on the mechanism research, the primary microglial cells were stimulated with LPS to activate the original protein complexes of NLRP3 so as to further explore the intrinsic mechanism by which DEX inhibited NLRP3 inflammasome. The results showed that DEX reduced NLRP3 expression in a dose-dependent manner, but had no effect on the expression of the components of NLRP3 inflammasome assembly, suggesting that DEX might be responsible for the degradation pathway of NLRP3 (Figures 5A,B). It was speculated that DEX might be responsible for the degradation pathway of NLRP3 to verify the effectiveness of two major degradation pathways (ubiquitin-proteasome and autophagy-lysosome pathways) in eukaryotes. The ubiquitin-proteasome and autophagy-lysosome pathways are two major degradation pathways in eukaryotes. They were blocked by treating the primary microglia with 3-MA and MG132, respectively. The immunoblotting analysis confirmed that the degradation of NLRP3 was inhibited by 3-MA, but not by MG132 (Figures 5C,D). These data indicated that DEX inhibited NLRP3 expression via promoting the autophagy pathway to degrade the protein. Simultaneously, DEX reduced NLRP3-mediated IL-1β secretion in an autophagy-dependent manner (Figures 5C,D). During autophagy induction, microtubule-associated protein 1 light chain 3 beta (MAP1LC3B; MAP1LC3B-I), a common marker of autophagosome formation, was covalently conjugated to phosphatidylethanolamine and converted into a fatty form (MAP1LC3B--II) on the elongated phagocyte membrane. Therefore, the increased conversion of MAP1LC3B-I into MAP1LC3B-II might serve as a sign of autophagosome formation. Next, the expression of MAP1LC3B was detected, revealing that DEX promoted autophagy and increased the production of MAP1LC3B-II slightly (Figures 5E,F). Sometimes, the decrease in MAP1LC3B-II levels was due to the rapid depletion of autophagy flux. This possibility was based on the accumulation of MAP1LC3B-II in the absence and presence of autophagy flux blockers such as Bafilomycin A1 (BafA1). DEX increased the accumulation of MAP1LC3B-II significantly in the presence of BafA1, indicating that it promoted an increase in the autophagy flux (Figure 5E). Next, the changes in the expression of ATG genes were compared between the control (CON) and DEX groups following LPS treatment. As shown in Figures 5G,H, DEX upregulated the LPS-stimulated reduction of the expression of autophagy related 5 (ATG5) and autophagy related 7 (ATG7). It also reduced the LPS-induced blockade of SQSTM1 protein and increased the conversion of MAP1LC3B-I into MAP1LC3B-II (Figures 5G,H).

On autophagy induction, red and green fluorescence co-localization was observed in the cells. In the late stage of autophagy, lysosomes and autophagosomes formed autophagolysosomes, intracellular pH decreased, GFP fluorescence decreased in acidic environments, and RFP was relatively stable. Hence, the two-point ratio of GFP–RFP could be used to evaluate the progress of the autophagy flow. Tandem fluorescent-mRFP-GFP-MAP1LC3B was transfected into primary microglial cells. In the present study, the green fluorescence of DEX-treated cells weakened, and the spots of red fluorescence increased. This effect was similar to that of the autophagy inducer rapamycin. BafA1 further inhibited the DEX-mediated reduction of red fluorescence of GFP (Figures 5I,J). Therefore, DEX promoted not only the formation of early autophagosomes in microglial cells but also the formation of autophagolysosomes in late stages. Taken together, these results showed that DEX promoted the degradation of NLRP3 and the NLRP3-mediated IL-1β secretion through the autophagy pathway, thus improving the inflammatory response.

### DEX Promoted Ubiquitination-Mediated Degradation of NLRP3

**FIGURE 6:**
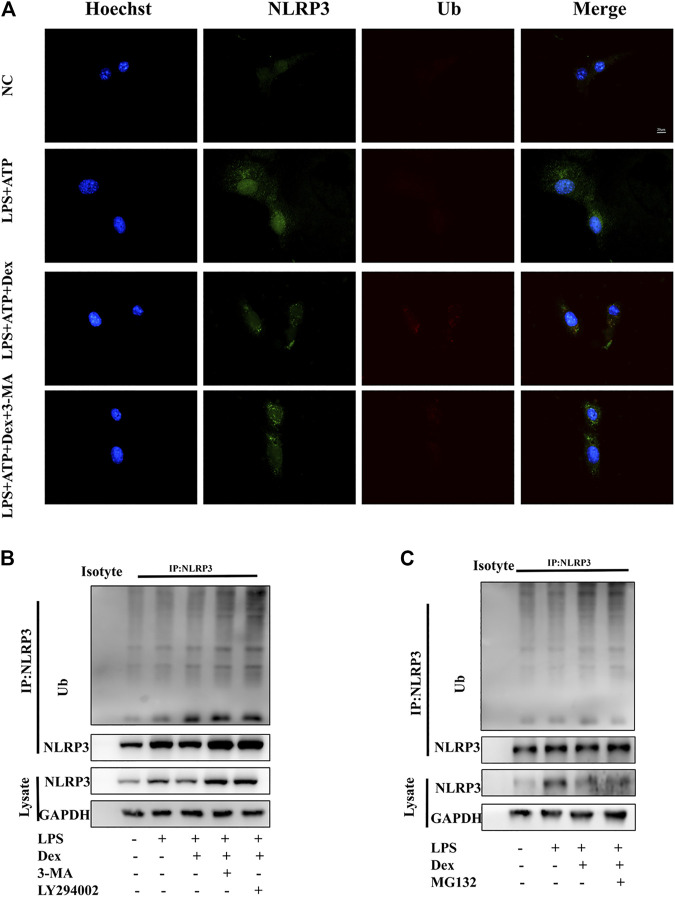
DEX promoting ubiquitination-mediated degradation of NLRP3. DEX promoted ubiquitination-mediated degradation of NLRP3. **(A)** Primary microglia cells were treated the same way as before. Cells were analyzed by NLRP3 and ubiquitin immunofluorescence. Scale bar = 20 μm. LPS-primed primary microglial were cultured with MG-132 (10 μM), 3-MA (5 mM) or, LY294002 (10 µM) for 1 h and then treated with DEX (10 μM) for 5 h. Immunoblotting analysis of Ub protein level in cell lysates immunoprecipitated with NLRP3 antibody **(B,C)**.

In the initial phase of autophagy, STSQTM1 (a receptor protein) is vital in the degradation of substrate proteins, which are linked to ubiquitin-modified substrates and then transported to autophagosomes for degradation. The results showed the involvement of SQSTM1 in the effect of DEX on NLRP3 degradation via the autophagy pathway. Similarly, attempts were made to investigate whether the ubiquitination degradation pathway was a key regulatory mechanism for the activation and assembly of NLRP3 inflammasomes. The experimental result in this part of the study confirmed that DEX-induced degradation of NLRP3 protein was regulated by SQSTM1-related autophagy signals. Considering the importance of ubiquitination for substrate protein degradation, the role of DEX in mediating the degradation of NLRP3 ubiquitination was analyzed. First, the immunofluorescence data revealed that LPS and ATP inhibited the fluorescence signal of ubiquitin protein in primary microglia; also, DEX enhanced the fluorescence signal of ubiquitin protein and inhibited the fluorescence signal of NLRP3 (Figure 6A). The ubiquitinated substrate was removed by autophagy via the proteasome pathway. Therefore, the present study further explored the mechanism of DEX-induced degradation of NLRP3. The DEX-induced degradation of NLRP3 was inhibited by autophagy inhibitor 3-MA and lysosomal inhibitor LY294002, resulting in the failure of degradation of ubiquitinated NLRP3 and the increase in its deposition in the cytoplasm (Figure 6B). In contrast, the ubiquitin-proteasome inhibitor MG132 had no significant effect on this process (Figure 6C). The results showed that DEX accelerated the degradation of NLRP3 protein by promoting the ubiquitin-mediated autophagy process.

## Discussion

**FIGURE 7:**
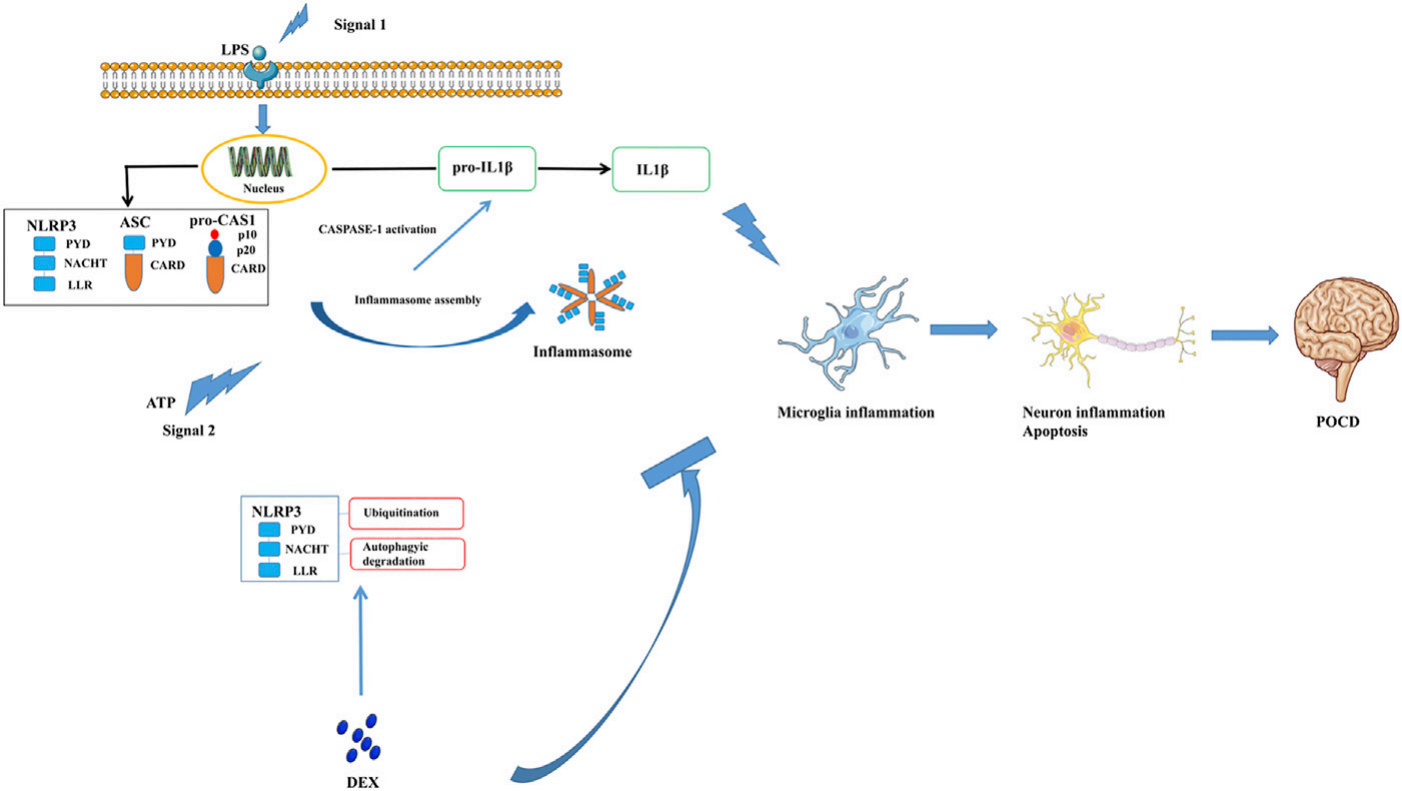
Dexmedetomidine, an α2 adrenergic receptor agonist, alleviates the cognitive function via blocking the activation and assembly of NLRP3 inflammasome and accelerated the degradation of NLRP3 through the ubiquitin-autophagy pathway. Consequently, neuroinflammation level induced by surgery was reduced, thenPND of mice has been improved.

This study confirmed that DEX mitigated NLRP3 inflammasome activation via autophagy-mediated microglial-mediated immunity, preventing long-lasting neuroinflammation and neuronal damage such as excessive CASP1 activation and IL-1β secretion, which eventually improved PND (Figure 7) through the autophagy pathway. In order to eliminate the influence of interfering factors in experimental animals on the mechanism research, we isolated primary microglia for *in vitro* experiments to study the deeper cellular mechanism. Its main anti-inflammatory mechanism was by accelerating the degradation of the inflammation-related molecule NLRP3 through the ubiquitin-autophagy pathway. Hence, autophagy-mediated microglial-mediated immunity focused on eliminating damage-associated molecular patterns, while preventing long-lasting neuroinflammation and neuronal damage, such as excessive IL-1β secretion, to improve postoperative cognitive impairment (Figure 7). These findings suggested the underlying mechanism and clinical effect of DEX on PND treatment.

An uninhibited early inflammatory process, including systemic metabolic inflammation and acute inflammation induced during surgical anesthesia, contributes to the perioperative cognitive function and the balance of brain homeostasis. One of the best examples of the clinical inflammatory response and postoperative cognitive impairment is in elderly individuals or people with metabolic diseases (Hermanides et al., 2018). Therefore, an urgent strategy is needed to target inflammatory responses and thus prevent postoperative cognitive impairment. The present study showed that the α2 receptor agonist DEX reduced the activation of NLRP3 through the ubiquitin-autophagy pathway, thereby mitigating the release of IL-1β.

Cognitive dysfunction is a common clinical manifestation of many neurodegenerative diseases and aging-related diseases. Increasing evidence showed that the abnormal activation of the immune system and the inflammation of the nervous system were the main factors triggering cognitive dysfunction (Forloni and Balducci, 2018; Low et al., 2019). Previous reports indicated that NLRP3 inflammasomes were associated with many neurodegenerative diseases, including PND (Feng et al., 2017; Liu et al., 2018; Perner et al., 2019). However, the underlying regulatory mechanisms are unclear. In the CNS, the activation of NLRP3 occurs in two classical steps: priming (the first signal) and activation (the second signal) (Mangan et al., 2018). The innate immune system recognizes risk-related and pathogen-related molecular patterns through pattern recognition receptors (Mangan et al., 2018). The first signal activates Toll-like receptor (TLR), IL-1β receptors, TNF-α receptors, and so forth, and then promotes the activation of the transcription of NLRP3, pro-IL-1β, and pro-IL-18. The second signal is activated under the stimulation of Ca^2+^ ion influx, which promotes the activation and assembly of NLRP3, ASC, and pro-CASP1 into activated forms, releases mature IL-18 and IL-1β, and mediates the inflammatory response (Heneka et al., 2018). Therefore, targeting the NLRP3–IL-1β signaling axis is an important way to treat the postoperative inflammatory response. Hence, mice or primary microglia were used to establish a model of PND or neuroinflammation, respectively.

The results showed that NLRP3 and AIM2 inflammasomes were activated in mice with surgery-induced PND; several other inflammasomes (NLRP1, NLRP2, and NLRC4) were not activated. The high level of IL-1β secreted by microglia was confirmed in postoperative animal models and clinical analyses (Wei et al., 2019). NLRP3 on microglia was significantly activated in mice with PND, suggesting that NLRP3 activation in microglia was associated with surgery-induced inflammation. These data were consistent with previous findings. Mamik and Power (2017) indicated that the NLRP3-mediated immune response prevented excessive inflammatory activation and long-term inflammatory response. Ising et al. (2019) found that the phosphorylation of Tau protein and cognitive function improved in NLRP3 knockout mice compared with wild-type mice. However, many targeted NLRP3 treatments were ineffective against cognitive impairment in various diseases (Athauda and Foltynie, 2015). In the CNS, the activation of NLRP3 involves two classical steps: activation of the first signal and activation of the second signal (Mangan et al., 2018). The innate immune system recognizes risk-related and pathogen-related molecular patterns through pattern recognition receptors (Mangan et al., 2018). The first signal activates TLR receptors, IL-1β receptors, TNF-α receptors, and so forth; promotes the activation of nuclear transcription factors; and mediates the expression of IL-18, IL-1β precursor protein, and NLRP3 protein. Under the stimulation of Ca^2+^ ion influx, the second signal is activated, which promotes the NLRP3 protein to recruit ASC through the PYD domain and then ASC to recruit PRO through the CARD domain. The second signal cleaves pro-CAS1 into an activated form, cleaves the precursor proteins of IL-18 and IL-1β, releases mature IL-18 and IL-1β, and mediates the inflammatory response (Heneka et al., 2018). The high level of IL-1β secreted by microglia has been confirmed in postoperative animal models and clinical analyses (Wei et al., 2019). Therefore, targeting the NLRP3–IL-1β signaling axis is an important way to treat a postoperative inflammatory response. Although the function of NLRP3 inflammasome has been widely reported, many of its functions are unknown. For example, the NLRP3-mediated immune response can prevent excessive inflammatory activation and long-term inflammatory response (Mamik and Power, 2017). Therefore, the role of NLRP3 in the development of acute and chronic inflammation is different. In addition, many targeted NLRP3 treatments are ineffective against cognitive impairment in many diseases (Athauda and Foltynie, 2015). However, in animal experiments, the phosphorylation of Tau protein and cognitive function improved in NLRP3 knockout mice compared with wild-type mice (Ising et al., 2019). However, the protective effect based on the gene knockout operation is difficult to achieve in clinical treatment. In addition, IL-1β-neutralizing antibodies and inhibitors of activation of CASP1 cannot completely improve inflammation. Therefore, finding out the mechanism regulating the activation of NLRP3 may be more effective compared with gene-controlled therapies.

Previous studies demonstrated that the α2 receptor agonist DEX was relieved by blocking the first signal of the activation of NLRP3 inflammasome in sepsis and acute kidney injury (Feng et al., 2019; Sun et al., 2019). However, given that the role of NLRP3 in the development of acute and chronic inflammation is different, the effect and mechanism of action of DEX on NLRP3 inflammasome in postoperative cognitive function are still unknown. The present study demonstrated that DEX reduced surgically induced activation of NLRP3 inflammasome and glial cells, as well as nerve damage, exhibiting neuroprotective effects in PND models.

The autophagy control system is related to the pathogenesis, such as mediating neurogenesis and regulating inflammatory response, in many neurodegenerative diseases such as PD and some metabolic diseases (Choi et al., 2020; Finkbeiner, 2020). Studies showed that NLRP3 inflammasome activity was negatively regulated by autophagy (Miyauchi et al., 2019; Marín-Aguilar et al., 2020). This study demonstrated that DEX reduced the activation of CASP1 and the release of mature IL-1β by reducing the assembly activation of NLRP3 both *in vivo* and *in vitro*, while the anti-inflammatory effect of DEX was blocked using 3-MA and BafA1 *in vitro*. The data indicated that the regulation of NLRP3 by autophagy reduced the damage caused by the inflammatory response. Furthermore, the present study found that DEX not only accelerated autophagy during the initiation phase but also contributed to autophagic flux integrity. Hence, it was confirmed that DEX reduced neuronal inflammation by accelerating the degradation of NPRP3 protein, rather than a decrease in “signaling IIˮ stimulation, resulting in the interruption of the assembly of the NLRP3–PYCARD–CASP1 complex. The findings of the present study were as follows. First, it confirmed that DEX suppressed the activation of CASP1 and the release of mature IL-1β by reducing the assembly activation of NLRP3 both *in vivo* and *in vitro*, while the anti-inflammatory effect of DEX was blocked using 3-MA and BafA1 *in vitro*. The data indicated that the regulation of NLRP3 by autophagy reduced the damage caused by the inflammatory response. Next, it found that DEX not only accelerated autophagy during the initiation phase but also contributed to autophagic flux integrity. However, the specific molecular mechanism underlying DEX-mediated autophagy to accelerate the degradation of NLRP3 needs further exploration.

Accumulating evidence emphasized the important relationship between autophagy, ubiquitination, and NLRP3. Ubiquitin ligase labels inflammatory substrates through poly-Ub chains, binds poly-Ub chains to the ubiquitin-binding associated protein domain of SQSTM1 protein, and guides the autophagy clearance of protein aggregates and organelle removal (Komatsu et al., 2012). The NLRP3 protein is recognized and degraded by ubiquitination, and is vital in the development of NLRP3 inflammasome–mediated inflammation (Baker et al., 2017). Subsequent evidence suggested that both dopamine and bile acids induced NLRP3 ubiquitination through the cyclic adenosine monophosphate-protein kinase A (cAMP-PKA) pathway (Yan et al., 2015), which is vital in suppressing inflammation. In addition, E3 ubiquitin ligase tripartite motif containing 31 subjected NLRP3 to ubiquitination and proteasome degradation (Song et al., 2016); another E3 ubiquitin ligase membrane–associated ring-CH-Typr finger 7 mediated the degradation of NLRP3 through K48-linked ubiquitination and autophagy (Yan et al., 2015). The data emphasized the important relationship between autophagy, ubiquitination, and NLRP3. The present study confirmed that DEX induced NLRP3 ubiquitination and degraded the protein through the autophagy pathway in primary microglia. After 3-MA, LY294002 and BafA1 were administered to the cells. After autophagy was inhibited, the protein was not successfully degraded. However, NLRP3 was degraded smoothly after the administration of proteasome inhibitor MG132, indicating that the degradation did not occur through the proteasome pathway.

In conclusion, this study provided strong evidence for DEX resistance to inflammation-mediated cognitive impairment induced by surgery. The main molecular mechanism was that DEX reduced NLRP3 expression through the ubiquitin-autophagy pathway and hindered the assembly and activation of the NLRP3 inflammasome, thereby minimizing the activation of downstream CASPA1 and the release of mature IL-1β. The findings might provide guidance for treating patients with inflammation-linked diseases, especially PND. The reasonable clinical use of DEX could also reduce postoperative cognitive impairment.

## Data Availability

The original contributions presented in the study are included in the article/Supplementary Material, further inquiries can be directed to the corresponding author.
